# Alpha-Lipoic Acid Attenuates Renal Injury in Rats with Obstructive Nephropathy

**DOI:** 10.1155/2013/138719

**Published:** 2013-10-03

**Authors:** Orawan Wongmekiat, Dolrawee Leelarungrayub, Kamthorn Thamprasert

**Affiliations:** ^1^Department of Physiology, Faculty of Medicine, Chiang Mai University, Chiang Mai 50200, Thailand; ^2^Clinical Biochemistry and Movement Sciences Research Group, Faculty of Associated Medical Sciences, Chiang Mai University, Chiang Mai 50200, Thailand; ^3^Department of Pathology, Faculty of Medicine, Chiang Mai University, Chiang Mai 50200, Thailand

## Abstract

This study was established to determine the possible protective effects of alpha-lipoic acid (ALA), a powerful antioxidant, on renal injury in obstructive nephropathy. Male Sprague-Dawley rats were assigned into sham-operated unilateral ureteral obstruction (UUO) and UUO treated with ALA groups. ALA 60 mg/kg was injected intraperitoneally 2 days before UUO induction and continued afterward for 7 days. Renal function, oxidative stress markers, nitric oxide, transforming growth factor-1 (TGF-**β**1), and histological changes were evaluated at the end of the experiment. Obstruction of the ureter resulted in renal dysfunction as indicated by significant increases in blood urea nitrogen and serum creatinine. Nonobstructed contralateral kidneys in all groups examined did not show any morphological or biochemical alterations. In untreated UUO group, the obstructed kidney developed marked hydronephrosis, leukocyte infiltration, and severe interstitial fibrosis. These functional and structural changes were associated with significant increases in tissue levels of malondialdehyde, nitric oxide, and TGF-**β**1 but decreases in reduced glutathione and total antioxidant capacity. Pretreatment with ALA significantly minimized all the changes elicited by ureteral obstruction. These findings demonstrate that ALA supplementation attenuates renal injury in rats with obstructive nephropathy and further suggest that oxidative stress inhibition is likely to be involved in the beneficial effects of this compound.

## 1. Introduction

Obstructive nephropathy remains an important cause of renal injury in both adults and children and may progress to chronic kidney disease and, eventually, end-stage renal disease [1, 2]. Despite timely surgical management of urinary tract obstruction, irreversible damage of the postobstructed kidney has been demonstrated [3]. Thus, relief of obstruction by itself may not be sufficient to improve the function and histopathology of the kidney but may require a concurrent pharmacological intervention.

Free radical generation with subsequent oxidative stress constitutes the mechanism of production and progression of numerous renal diseases, including obstructive nephropathy [2, 4, 5]. Alpha-lipoic acid (ALA) is a naturally occurring dithiol compound that functions as an essential cofactor for mitochondrial bioenergetic enzymes [6]. It has also been recognized as a powerful antioxidant capable of prevention or treatment of many diseases associated with oxidative stress, such as diabetes, chronic liver diseases, and neurodegenerative processes [7–10]. Recently, ALA has been reported to protect the renal tissue from ischemia-reperfusion injury and various drug-induced toxicities [7, 11–13]. The question therefore arises whether the obstructed kidney may benefit from ALA treatment.

The present study was established to test the possible renoprotective effect of ALA, via its antioxidant properties, in rats with obstructive nephropathy. The investigation was performed using unilateral ureteral obstruction (UUO) model because it is a normotensive nonuremic disorder, without any apparent immune or toxic renal insult, that mimics the complex pathophysiology of human chronic obstructive nephropathy in an accelerated manner [1, 4].

## 2. Materials and Methods

### 2.1. Drugs and Chemicals

All chemicals used in this study, or otherwise stated, were of analytical grade and were purchased from Sigma Chemical Co. (St Louis, MO, USA).

### 2.2. Animals

Male Sprague-Dawley rats (200–220 g) were kept under a 12 h light-dark cycle at 25°C and allowed free access to food and water. The animals were acclimatized for one week before starting the experiment. All procedures were conducted in accordance with the guidelines for care and use of laboratory animals and were approved by the Animal Care Committee at the Faculty of Medicine, Chiang Mai University, Thailand.

### 2.3. Induction of UUO

UUO was performed under gaseous anesthesia with a mixture of isoflurane (Abbott, IL, USA) and oxygen. The left kidney was exposed through a flank incision, and a double ligature (0.5 cm apart) with 4–0 silk was placed on the left ureter approximately 1 cm below the renal hilum and cut between the ligatures to prevent retrograde infection. For sham-operated group, rats underwent identical anesthetic procedure followed by surgical intervention of a comparable duration without the final step of ureteral ligation. All surgical procedures were carried out under aseptic technique.

### 2.4. Experimental Design

Rats were allocated into 3 groups as follows: (1) sham group, which was subjected to sham-operation and injected intraperitoneally with vehicle, (2) UUO group, which was subjected to UUO and vehicle injection, (3) UUO + ALA group, which was subjected to UUO and injected intraperitoneally with 60 mg/kg ALA. This dose was chosen as it has been shown to be the no-observed-adverse-effect level (NOAEL) [14]. All injections were started 2 days before the operation and continued afterward for 7 days. At the end of experiment, blood sample was collected, and both kidneys were removed under pentobarbital (Thiopental, ICN a.s., Roztoky, Czech Republic) anesthesia (80 mg/kg, i.p.). Parts of the obstructed as well as contralateral kidneys were fixed in 10% neutral buffered formalin for histopathological evaluation. The remaining kidney tissues were snap-frozen in liquid nitrogen and kept at −80°C for biochemical studies.

### 2.5. Biochemical Studies

#### 2.5.1. Renal Function Test

Serum sample was assayed for urea nitrogen and creatinine using automatic analyzer (Beckman Coulter, Inc., CA, USA).

#### 2.5.2. Preparation of Tissue Homogenate

The kidney tissue was homogenized in an ice-cold phosphate buffered saline, pH 7.4 using a Potter Elvehjem homogenizer (Wheaton Science, Millville, NJ). The homogenate was centrifuged at 10000 g for 10 min at 4°C, and the supernatant was collected for determinations of malondialdehyde (MDA), reduced glutathione (GSH), total antioxidant capacity (TAC), nitric oxide (NO), and transforming growth factor-*β*1 (TGF-*β*1).

#### 2.5.3. Determination of Malondialdehyde

MDA was estimated by measuring thiobarbituric acid reactive substances as described by Ohkawa et al. [15]. Briefly, the reaction mixture consisted of 0.1 mL of supernatant, 1 mL of 100% trichloroacetic acid in 0.6 M HCl, 0.5 mL of 0.9% NaCl, and 0.2 mL of 0.12 M thiobarbituric acid. The mixture was heated at 95°C for 30 min and, after cooling with tap water, was centrifuged at 10000 g for 5 min at 4°C. The absorbance of the organic layer was read at 532 nm. The MDA concentration was quantified from a standard curve of 1,1,3,3-tetramethoxypropane and expressed as mM/g kidney wt.

#### 2.5.4. Determination of Reduced Glutathione

GSH concentration was determined spectrophotometrically according to the method of Beutler [16]. A 0.4 mL of supernatant from tissue homogenate was precipitated with precipitant containing 0.2 g EDTA, 1.67 g metaphosphoric acid, and 30 g NaCl in 100 mL of dH_2_O. The sample was centrifuged at 10000 g for 10 min at 4°C. The assay mixture contained 0.2 mL of the filtered aliquot, 0.4 mL of 0.3 M phosphate buffer (pH 8.0), and 0.4 mL of 5-5′-dithiobis-2-nitrobenzoic acid (40 mg in 100 mL dH_2_O containing 1% sodium citrate). The absorbance was read at 412 nm after incubation within 5 min. The concentration of GSH was determined from a GSH standard curve and expressed as mM/g kidney wt.

#### 2.5.5. Determination of Total Antioxidant Capacity

TAC was determined by antioxidant assay kit obtained from Cayman Chemical Company (Ann Arbor, MI, USA) according to the manufacturer's instruction. The assay was based on the ability of antioxidants in the sample to inhibit ABTS (2,2′-azino-di-[3-ethylbenzthiazoline sulfonate]) oxidation. The result was expressed as mM of Trolox equivalents/g kidney wt.

#### 2.5.6. Determination of Nitric Oxide

Total NO levels were evaluated using QuantiChrom nitric oxide assay kit (Bioassay Systems, Hayward, CA, USA) according to the manufacturer's instruction, which is based on the reduction of nitrate to nitrite by Griess method. The amounts of total nitrite were determined as a measure of NO generation and expressed as *μ*M/g kidney wt.

#### 2.5.7. Determination of Transforming Growth Factor-Beta 1

TGF-*β*1 levels were assayed using commercial enzyme-linked immunosorbent assay kit (R&D Systems, MN, USA) at 450 nm according to the manufacturer's instruction. Results were expressed as pg/g kidney wt.

### 2.6. Histopathological Studies

The fixed kidney tissues were processed in graded alcohol and xylene and then embedded in paraffin. Sections of 4 mm thickness were deparaffinized, hydrated, and stained with hematoxylin and eosin (H&E) and Masson's trichrome for morphological studies. The severity of injury was graded by an experienced pathologist who was unaware of the treatment groups using a method previously described by Demirbilek et al. [17].

H&E stained-kidney sections were used to evaluate leukocyte infiltration. Briefly, the widening of interstitial spaces with focal leukocyte infiltration was assessed in five sections prepared from each kidney sample. For each section, ten randomly selected separate nonoverlapping microscopic fields were examined under light microscope at 400x magnification. The number of leukocytes was counted and averaged to yield the number of leukocytes for each kidney section, after which the mean values from five kidney sections of each animal were calculated.

The extent of interstitial fibrosis was estimated from kidney tissues stained with Masson's trichrome. Briefly, the fibrotic area in the interstitium, stained blue by Masson's trichrome, was picked up on the digital images under high magnification (400x) using a computer-aided manipulator (Image analyzer with Carl Zeiss Axiovision LE, free software analysis). The fibrotic area relative to the total area of the field was analyzed and calculated as a percentage. The average score from ten non-overlapping fields per section was determined, thereafter; the mean scores from five sections per animal were calculated.

### 2.7. Statistical Analysis

Data are presented as mean ± SEM. Comparisons were performed by one way ANOVA followed by Bonferroni's Dunn post-hoc test using the SPSS 16.0 software (SPSS Inc., Chicago, IL, USA). *P* < 0.05 was considered statistically significant.

## 3. Results

### 3.1. Kidney Weight/Body Weight Ratio

In UUO group, obstruction of the ureter resulted in ipsilateral hydronephrosis as demonstrated by higher kidney weight/body weight ratio than its corresponding contralateral kidney as well as that of the sham group (all *P* < 0.001) (Table 1). UUO rats-pretreated with ALA significantly minimized this condition (*P* < 0.01), but the value of kidney weight/body weight ratio obtained in this group remained slightly higher (*P* < 0.05) than that recorded from the sham control.

### 3.2. Renal Function Test

Blood urea nitrogen and serum creatinine were significantly increased (all *P* < 0.001) in the untreated UUO group compared to the sham group (Table 2). These elevations were significantly reduced (all *P* < 0.001) upon ALA treatment. However, the level of blood urea nitrogen detected from the ALA-treated UUO group remained slightly higher (*P* < 0.01) than that in the sham.

### 3.3. MDA, GSH, and TAC in the Kidney

**Figure 1:**
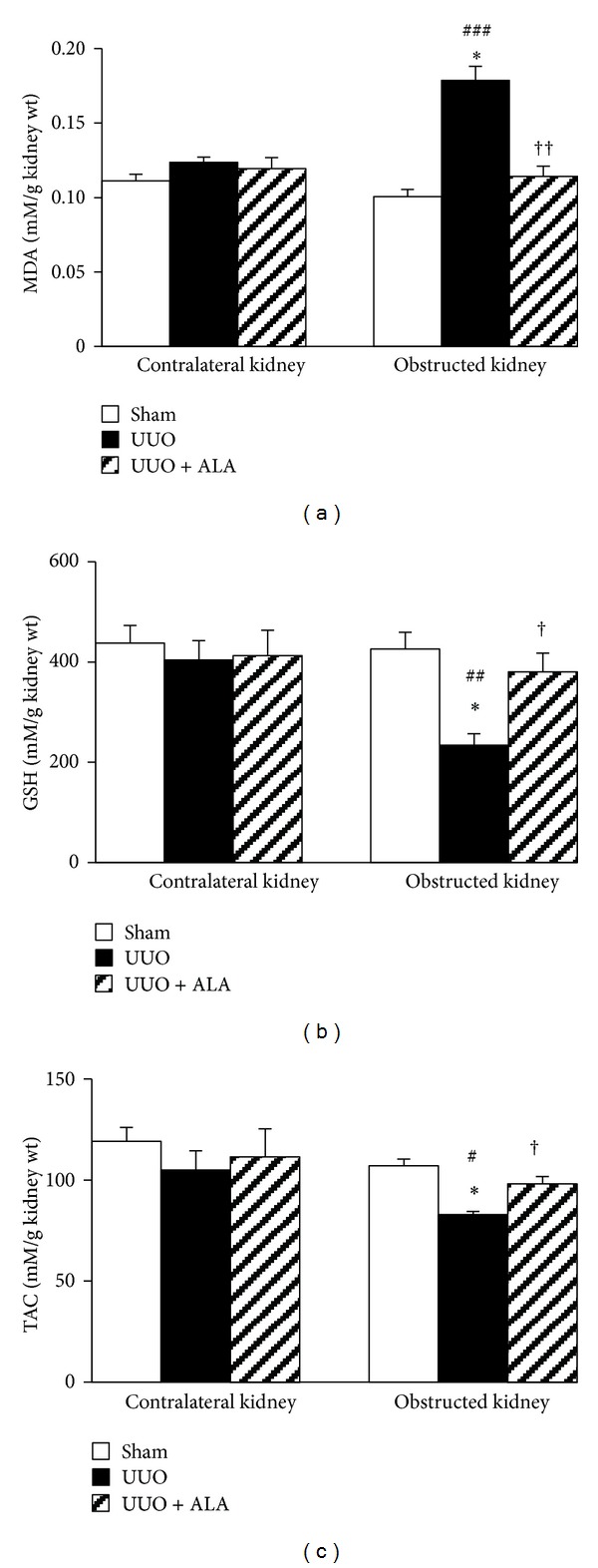
The effects of unilateral ureteral obstruction (UUO) and alpha-lipoic acid (ALA) on (a) malondialdehyde (MDA), (b) reduced glutathione (GSH), and (c) total antioxidant activity (TAC). Values are mean ± SEM from 10 rats in each group. *P < 0.001 versus sham, ^†^ *P* < 0.01, ^††^ *P* < 0.001 versus UUO within the same kidney. ^#^ *P* < 0.05, ^##^ *P* < 0.01, ^###^ *P* < 0.001 versus corresponding contralateral kidney.

The levels of MDA, GSH, and TAC in the nonobstructed contralateral kidney were very similar among the groups (Figure 1). On the contrary, a significant increase in MDA (Figure 1(a)), but decrease in GSH (Figure 1(b)) and TAC, (Figure 1(c)) were observed in the obstructed kidney of untreated UUO rats compared to the ipsilateral sham control (all *P* < 0.001). Supplementation of the UUO rats with ALA restored all the changes in MDA, GSH, and TAC caused by UUO to the values that were comparable to those of the sham rats.

### 3.4. NO and TGF-*β*1 in the Kidney

**Figure 2:**
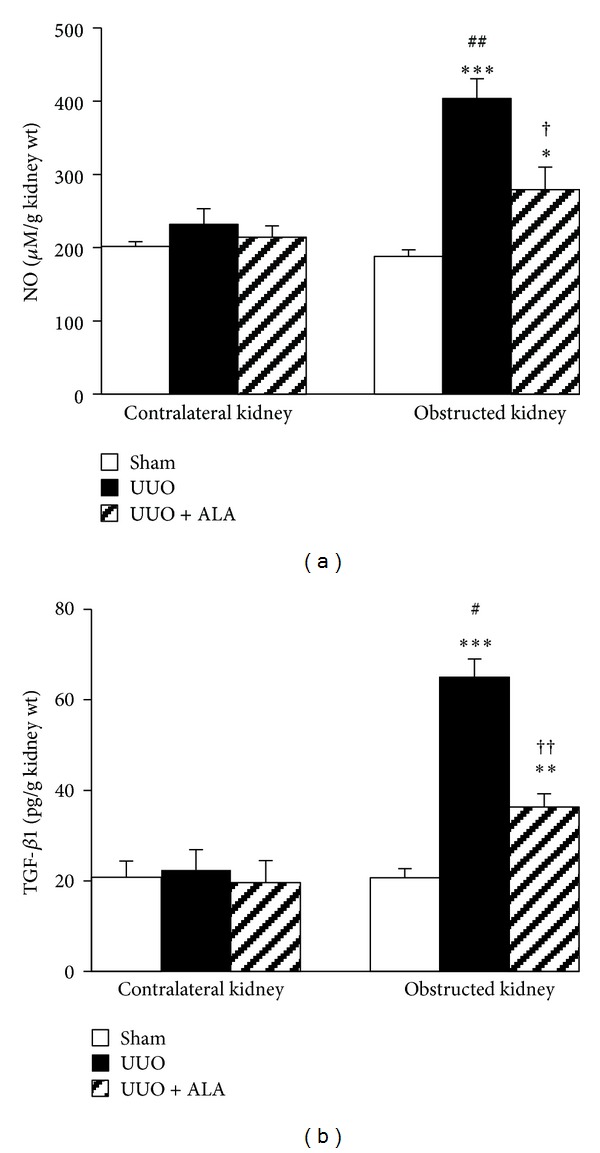
Effects of unilateral ureteral obstruction (UUO) and alpha-lipoic acid (ALA) on (a) nitric oxide (NO) and (b) transforming growth factor-*β*1 (TGF-*β*1) levels. Values are mean ± SEM from 10 rats in each group. *P < 0.05, **P < 0.01, ***P < 0.001 versus sham, ^†^ *P* < 0.01, ^††^ *P* < 0.001 versus UUO within the same kidney. ^#^ *P* < 0.01, ^##^ *P* < 0.001 versus corresponding contralateral kidney.

As shown in Figure 2(a), induction of UUO caused an ipsilateral increase in the level of NO compared with sham operation (*P* < 0.001). This increase was significantly blunted, although, to some extent, by coadministration of ALA (*P* < 0.01). TGF-*β*1 level in the obstructed kidney of UUO group was also elevated approximately 3-fold from the value obtained in the sham group (*P* < 0.001), and ALA treatment was partially, but significantly (*P* < 0.001), capable of preventing this elevation by 44% (Figure 2(b)). In the non-obstructed contralateral kidney, however, there were no significant differences of both NO and TGF-*β*1 levels in all groups studied.

### 3.5. Histopathological Changes

**Figure 3:**
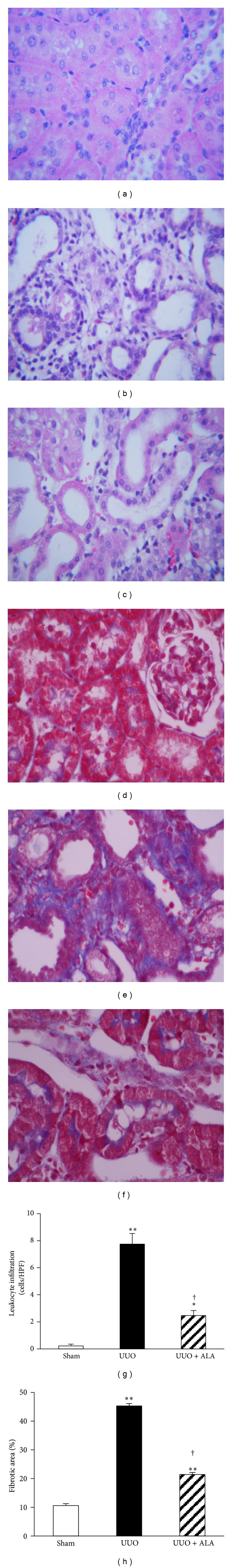
Effects of unilateral ureteral obstruction (UUO) and alpha-lipoic acid (ALA) on renal histopathological changes in the obstructed kidney from sham ((a), H&E 40x; (d), Masson's trichrome 40x) showing normal tubules and peritubular stromal tissues, UUO group ((b), H&E 40x; (e), Masson's trichrome 40x) showing dilated tubules, marked interstitial infiltration with polymorphonuclear leukocytes and severe interstitial fibrosis, UUO + ALA group ((c), H&E 40x; (f), Masson's trichrome 40x) showing mild leukocyte infiltration and less fibroblastic proliferation. The degree of leukocyte infiltration (g) and fibrotic area (h) are shown as mean ± SEM from 10 rats in each group. *P < 0.01, **P < 0.001 versus sham. ^†^ *P* < 0.001 versus UUO.

The non-obstructed contralateral kidneys of all experimental groups showed essentially normal histology and, therefore, were excluded from further analysis (data not shown). Ipsilateral obstructed kidney sections from sham-operated rats (Figures 3(a) and 3(d)) also appeared normal, whereas those from untreated-UUO rats (Figures 3(b) and 3(e)) exhibited severe and diffuse tubular dilatation in both cortex and medulla with focal vacuolar degeneration and pressure atrophy. Widening of fibrovascular stroma with prominently fibroblastic proliferation and predominately polymorphonuclear leukocyte (PMN) infiltration was obvious in the obstructed kidney. Hyaline collagen substance was observed focally. The foci of PMN and nuclear debris aggregation were also demonstrated in the submucosal stroma of the dilated calyces. These structural changes were markedly improved, when UUO was accompanied by ALA treatment (Figures 3(c) and 3(f)). The extent of leukocyte infiltration (Figure 3(g)) and fibrotic area (Figure 3(h)) was reduced almost 68% and 52%, respectively, in UUO rats treated with ALA compared to the untreated rats (both *P* < 0.001).

## 4. Discussion

The current investigation addresses the possible renoprotection by ALA in the setting of UUO. The results show that obstruction of the ureter leads to renal inflammation and subsequent fibrosis, and ALA, at least in part, restores renal integrity by blocking oxidative stress related TGF-*β*1-induced fibrogenesis.

Renal fibrosis is the common end point of virtually all progressive kidney diseases [18, 19]. The degree of renal fibrosis is also considered a reliable predictor of renal prognosis [19]. Various cytokines, chemokines, and growth factors are involved in the development of fibrosis; however, TGF-*β*1 is considered to be the most potent and ubiquitous profibrogenic cytokine [18–20]. Studies have shown that TGF-*β*1 promotes fibroblast proliferation and their further differentiation to become myofibroblasts, the key players in fibrogenesis [18, 19, 21]. In addition, increased TGF-*β* mRNA and/or protein expression has been reported in various fibrotic diseases in multiple organ systems, including the kidney [18]. In the present study, a significant increase in TGF-*β*1 level was observed following ureteral obstruction. Histological examination also demonstrated hydronephrosis as well as the presence of fibroblast and several renal and infiltrating cell types, particularly polymorphonuclear leukocyte, in the interstitium. These findings not only confirm the development of renal inflammation and fibrosis in the current investigation but also provide further support to the view of TGF-*β*1-mediated fibrogenesis in obstructive kidney disease.

Although TGF-*β*1 is undoubtedly contributed to obstruction-induced fibrosis, it is normally secreted as a latent procytokine complex that needs activation before signaling through TGF-*β* receptor and mediating fibrogenic effects [18, 20]. In the present study, elevation of TGF-*β*1 is accompanied by the increased MDA and NO levels, together with depleted GSH concentration and total antioxidant capacity, indicating the interplay between oxidative stress and TGF-*β*1-induced fibrogenesis. This is consistent with several reports suggesting a vicious loop between ROS and TGF-*β*. Studies have demonstrated that TGF-*β* stimulates the production of ROS, whereas ROS activate/induce TGF-*β* gene expression and mediate many of the fibrogenic effects of TGF-*β* [18]. Increased free radical formation (ROS/RNS), lipid peroxidation, oxidative protein, and DNA damage together with decreased antioxidant defenses have all been observed in fibrotic kidney in various experimental models, including UUO [2, 4, 5, 17, 22]. Most importantly, evidence exists that these oxidant-related molecules were detectable only few minutes after the onset of UUO, indicating that oxidative stress is likely the first stressor in the cascade of these complex disorders [2]. Taken together, the findings reported herein provide additional evidence to reinforce the critical role of oxidative stress-mediated fibrogenesis induced by TGF-*β*1 in the pathogenesis of tubulointerstitial injury associated with obstructive nephropathy.

The present investigation revealed that pretreatment with ALA retarded tubulointerstitial fibrosis caused by ureteral obstruction. ALA is recognized as a universal antioxidant capable of scavenging free radicals, chelating metals, regenerating endogenous antioxidants, and modulating various signal transduction pathways [6, 8, 9, 14]. It is unique among antioxidants in its ability to display antioxidant properties in both oxidized and reduced forms as well as in both lipid and aqueous environments [6, 8, 9]. The therapeutic potential of ALA has been demonstrated in a variety of disorders linked to oxidative stress and inflammation in diverse organ systems, including the kidney [6, 7, 11–13]. In the present study, the reduction of oxidative product MDA, the restoration of GSH, and the maintenance of antioxidant capacity were apparent in the obstructed kidney treated with ALA. Based on these pieces of evidence, it is assumed that the antioxidant properties of ALA may underlie its renal benefits observed in this study. Previous research has shown that activation of the redox-sensitive nuclear transcription factor (NF-*κ*B) indirectly controls TGF-*β*1 expression by regulating the promoter of transglutaminase, which is an activator of latent TGF-*β*1 [23]. As a result of maintaining redox balance, ALA may impede oxidative stress-induced NF-*κ*B activation and, thus, reduce obstruction-induced fibrosis. Alternatively, ALA may suppress the activation of NF-*κ*B independent of its antioxidant as recent publication has demonstrated the ability of ALA to prevent the inhibitor-kappaB-*α* (I*κ*B*α*) degradation and NF-*κ*B dependent gene expression by directly inhibiting the activity of I*κ*B kinase-2 [24].

NO derived from inducible nitric oxide synthase (iNOS) has also been proposed to play an important role in tubulointerstitial inflammation associated with obstructive nephropathy [1, 25]. Increased iNOS activity and expression have previously been reported in obstructed kidney [1, 25, 26]. It has been suggested that the synergy of iNOS and proinflammatory cytokines such as TGF-*β*1 may lead to the accumulation of extracellular matrix (ECM) in the kidney and thereby cause tubular atrophy and interstitial fibrosis [1, 25]. In the present study, obstruction of ureter caused an inflammatory response as shown by the rise in NO levels as well as a dramatic PMN infiltration in the interstitium, and these observations were ameliorated by ALA treatment. Since ALA has been shown to inhibit iNOS expression [7, 11, 27], attenuation of iNOS-generating NO may be another component of the beneficial effect of ALA on the amelioration of the tubulointerstitial fibrosis caused by ureteral obstruction.

## 5. Conclusion

The present study reveals a promising role for ALA to ameliorate UUO-induced renal injury possibly through its antioxidant properties. The finding suggests that ALA may be beneficial as adjunctive therapy to improve the surgical outcome of obstructive nephropathy. However, the possibility of reducing obstructive damage by pretreatment does not seem convincing in the clinical situations, and, thus, further studies are necessary to prove whether ALA maintains its beneficial effects also when administered after the obstructive event as well as after relief of the obstruction. As renal damage continued after the relief of UUO, long-term evaluations of the impacts of ALA on the recovery of renal function either after temporary or chronic obstruction may also be essential in validating the effectiveness of this compound before transferring to the clinical practice.

## Figures and Tables

**Table 1:** Kidney weight/body weight ratio.

Group	Kidney weight/body weight (×100)	
	Contralateral kidney	Obstructed kidney
Sham	0.44 ± 0.02	0.42 ± 0.02
UUO	0.49 ± 0.02	0.63 ± 0.02^#∗∗^
UUO + ALA	0.48 ± 0.02	0.52 ± 0.03^∗†^

Values are mean ± SEM from 10 rats in each group. UUO: unilateral ureteral obstruction, UUO + ALA: unilateral ureteral obstruction plus alpha-lipoic acid, ^*#*^ *P* < 0.001 versus contralateral kidney within the group, *P < 0.05, **P < 0.001 versus sham, ^†^ *P* < 0.01 versus UUO.

**Table 2:** Blood urea nitrogen and serum creatinine.

Group	Blood urea nitrogen (mg/dL)	Serum creatinine (mg/dL)
Sham	22.87 ± 0.59	0.49 ± 0.03
UUO	33.13 ± 0.51**	0.98 ± 0.09**
UUO + ALA	26.19 ± 0.51^∗†^	0.62 ± 0.03^†^

Values are mean ± SEM from 10 rats in each group. UUO: unilateral ureteral obstruction, UUO + ALA: unilateral ureteral obstruction plus alpha-lipoic acid, *P < 0.01, **P < 0.001 versus sham, ^†^ *P* < 0.001 versus UUO.
